# Nanocarriers for the Delivery of Neuroprotective Agents in the Treatment of Ocular Neurodegenerative Diseases

**DOI:** 10.3390/pharmaceutics15030837

**Published:** 2023-03-03

**Authors:** Chirag Patel, Sonal Pande, Vrunda Sagathia, Ketan Ranch, Jayesh Beladiya, Sai H. S. Boddu, Shery Jacob, Moawia M. Al-Tabakha, Nageeb Hassan, Moyad Shahwan

**Affiliations:** 1 Department of Pharmacology, L. M. College of Pharmacy, Ahmedabad 380009, India; 2 Department of Pharmaceutics, L. M. College of Pharmacy, Ahmedabad 380009, India; 3 Department of Pharmaceutical Sciences, College of Pharmacy and Health Sciences, Ajman University, Ajman P.O. Box 346, United Arab Emirates; 4 Center of Medical and Bio-Allied Health Sciences Research, Ajman University, Ajman P.O. Box 346, United Arab Emirates; 5 Department of Pharmaceutical Sciences, College of Pharmacy, Gulf Medical University, Ajman P.O. Box 4184, United Arab Emirates; 6 Department of Clinical Sciences, College of Pharmacy & Health Science, Ajman University, Ajman P.O. Box 346, United Arab Emirates

**Keywords:** nanocarriers, ocular neurodegenerative diseases, diabetic retinopathy, retinal ganglion cell disease

## Abstract

Retinal neurodegeneration is considered an early event in the pathogenesis of several ocular diseases, such as diabetic retinopathy, age-related macular degeneration, and glaucoma. At present, there is no definitive treatment to prevent the progression or reversal of vision loss caused by photoreceptor degeneration and the death of retinal ganglion cells. Neuroprotective approaches are being developed to increase the life expectancy of neurons by maintaining their shape/function and thus prevent the loss of vision and blindness. A successful neuroprotective approach could prolong patients’ vision functioning and quality of life. Conventional pharmaceutical technologies have been investigated for delivering ocular medications; however, the distinctive structural characteristics of the eye and the physiological ocular barriers restrict the efficient delivery of drugs. Recent developments in bio-adhesive in situ gelling systems and nanotechnology-based targeted/sustained drug delivery systems are receiving a lot of attention. This review summarizes the putative mechanism, pharmacokinetics, and mode of administration of neuroprotective drugs used to treat ocular disorders. Additionally, this review focuses on cutting-edge nanocarriers that demonstrated promising results in treating ocular neurodegenerative diseases.

## 1. Introduction

**Figure 1:**
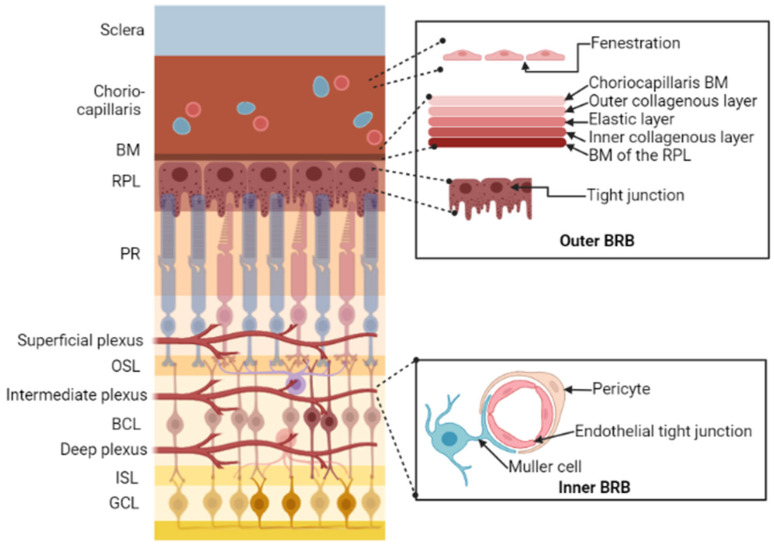
Schematic drawing of the blood-retinal barrier (BRB) structure. The outer BRB protects the outer retina from circulation derived from choriocapillaris, which has numerous fenestrations in its endothelium. Drug molecules enter the neuroretina through a five-layer structure called Bruch’s Membrane (BM), that provides a size-selective barrier and the retinal pigment layer (RPL), which is characterized by tight connections. The inner BRB protects inner retina from the retinal vasculature, which consists of a deep, intermediate, and superficial plexus; the endothelial cells of the retinal arteries constitute an efficient barrier due to the existence of tight connections among them [12]. PR, Photoreceptor; OSL, Outer Synaptic layer; BCL, Bipolar cell layer; ISL, Internal Synaptic layer; GCL, Ganglion cell layer. Image was created in BioRender.com.

The eye is a complex human body structure and is often considered an anatomic extension of the brain. The retina and optic nerve especially are considered parts of the brain. The retina is formed embryonically from neural tissue and the optic nerve connects the brain to the retina. The retina is a complex transparent tissue consisting of several layers of cells, including the primary light-sensing photoreceptor cells. Photoreceptors receive light input from the environment and pass it on to the retinal ganglionic cells (RGC), which convert the visual stimuli into electric impulses and send them to the brain via the optic nerve as a part of the visual process [1,2]. RGC and neurons have the same anatomical structures but different morphologies. Both are composed of a cell body, dendrites, and axons. RGCs axons create the optic nerve, which, like all nerves, is enclosed in myelin and meninges [3]. The blood–brain barrier (BBB) is present in the CNS, while the blood-retinal barrier (BRB) is present in the eye. A schematic drawing of the BRB structure is depicted in Figure 1. Both the RGCs and neurons do not multiply, except for a small number of progenitors. Therefore, RGC damage caused by degeneration or immune-mediated inflammation is irreversible and can result in a variety of eye diseases [4]. The common pathophysiologic pathway of these diseases include metabolic stress such as hypoxia, exposure to increased free radicals, mechanical compression, and specifically photooxidative damage by (mainly blue) light passing through the retina, resulting in an insufficient supply of nutrients to the respective target structures (optic nerve head, retina, and retinal pigmented epithelium). During metabolic stress, glutamate is released, initiating the death of neurones containing ionotropic glutamate (N-methyl-D-aspartat, NMDA) receptors present on ganglion cells, and a specific type of amacrine cells [5,6]. Further, oxidative stress is widely thought to hasten the course of AMD. The fundamentally high arterial O_2_ tension environment, radical generation in phototransduction, blue light damage, photooxidative lipofuscin-containing A2E buildup in the RPE, and loss of cellular antioxidant potential together contribute to oxidative stress in eye illness [7]. Recent studies have also found that aggressive glial cells target neurons and reduce antioxidant capacity inside neurons in some types of glaucoma [8]. These ocular disorders primarily affect the elderly population but can sometimes affect younger persons and result in irreversible blindness or diminished, degraded vision [9,10,11]. Blindness and reduced visual acuity are linked to lower quality of life measurements because they limit social contacts and independence and may result in job loss and depression. 

Several approaches, including gene therapy, stem cell therapy, pharmacological interventions, and target discovery through genomic research, have the potential for improving ocular impairment centered on tissue repair or regeneration [13]. Pharamcological interventions such as timolol, betaxolol, levobunolol, carteolol, brimonidine, and apraclonidine were used for reduction of intraocular pressure (IOP) and decrease aqueous humor production. Likewise, ranibizumab, aflibercept, and bevacizumab inhibit the biological activity of VEGF and reduce blood vessel growth [14]. There is currently no effective therapy or strategy to modify the disease progression and reverse vision loss caused by photoreceptor degeneration, loss of visual signal transmission to the brain, or death of RGCs. Recently, a wide range of therapies known as “neuroprotective approaches” have been developed to increase the life of neurons by maintaining their shape and function, preventing the vision loss and blindness [15]. Successful neuroprotective techniques could prolong an individual’s functioning vision over years or even decades, permitting them to retain their independence, quality of life, and opportunity to manage. Conventional pharmaceutical technologies have been investigated for delivering drugs to essential eye structures. However, the distinctive structural characteristics of the eye and the physiological ocular barriers present significant obstacles to the efficient delivery of the drugs at the required site [16]. For tackling these obstacles, several technologies, such as bioadhesive in situ gelling systems and nanotechnology-based drug delivery systems, are being used for targeted and sustained delivery of drugs to the anterior and posterior segments of the eye [17,18,19,20]. As compared to published reviews, this work summarizes various neurodegenerative diseases, putative mechanisms, pharmacokinetics, and mode of administration of neuroprotective drugs used in the treatment of ocular disorders. Additionally, this review focuses on cutting-edge nanocarriers which demonstrated effectiveness in treating ocular diseases.

## 2. Methodology

A thorough literature search was performed on different databases including Cochrane, PubMed/Medline, clinical trials.gov, and Google Scholar using the possible pairs of key words such as “neurodegenerative ocular disease”, “interventions for ocular disease”, “herbs proven for ocular neurodegenerative”, “neuroprotective agents for ocular”, “ocular barriers”, “physicochemical parameters”, “nanocarrier systems”. The studies were selected as per the same criteria by forward/backward search. Studies related to formulation, mechanism of disease induction, animal studies, review articles related to neuroprotective agents were mainly focused. Articles were sorted by relevance of recent years and more articles were identified through cross-references from the screened studies. 

## 3. Neurodegenerative Diseases Affecting the Eye

Age-related ocular neurodegenerative diseases mainly included diabetic retinopathy, retinal ganglion cell disease, and photoreceptor degenerative disease.

### 3.1. Degenerative Disease of the Photoreceptors

The photoreceptors are the structures that transform light energy into visual signals. Retinitis pigmentosa (RP) and age-related macular degeneration (AMD) are the two primary retinal disorders characterized by photoreceptor degradation [21,22]. Over 250 different gene mutations or other problems in photoreceptor or retinal pigment epithelial cells have been linked to the rare family of inherited retinal illnesses known as RP [23]. These deficiencies cause photoreceptor degeneration. Two different types of therapy are being investigated for RP. The first is gene therapy, which mainly focuses on vision restoration or residual vision activation by replacing components of the visual circuit, such as defective genes [24,25]. However, gene therapy is not precise concerning vision restoration. Although gene therapy may prevent deterioration of the vision, safety concerns limit gene therapy to people with advanced forms of inherited retinal illness [26]. Second, neuroprotective agents are being used to maintain and preserve the visual function of patients for many years [27]. To prevent the progression of vision loss to blindness, neuroprotective agents may be used after the initial detection of vision loss [28]. Basic fibroblast-derived growth factor (bFGF), brain-derived neurotrophic factor (BDNF), cardiotrophin-1, nerve growth factor (NGF), fibroblast growth factor (FGF), and CNTF are among the substances that decreased photoreceptor loss in animal models [29,30]. 

Central vision is largely affected by AMD, which is characterized by the thickening of Bruch’s membrane as a result of lipid and the protein accumulation and the appearance of extracellular deposits (also known as drusen) between the RPE and Bruch’s membrane. Drusen build-up, localized atrophy (regional loss of the choroid, RPE, and retinal neurons) and/or neovascularization develop as the condition worsens [31,32]. According to Bhutto and Lutty (2012), complement factor H mutations play an important role in the loss of the RPE or choroid vasculature before the onset of photoreceptor mortality [33]. Age, smoking, sunlight exposure, and genetic variables causing complement dysregulation are considered to be the risk factors for AMD [34]. There are two types of AMD, dry AMD and wet AMD. Wet AMD characteristics include retinal vascular anomalies, such as vessel leakage and angiogenesis [35]. Age-Related Eye Condition Study (AREDS) formula consisting of oral supplements including vitamin C, vitamin E, beta-carotene, zinc oxide, and cupric oxide have shown beneficial neuroprotective properties [36]. These agents have reduced the progression of intermediate and advanced AMD by 25% [37]. Anti-vascular endothelial growth factor (anti-VEGF) injections and laser therapy are additional methods of treating wet AMD [38]. More neuroprotective approaches should be developed in order to curb the increasing prevalence of AMD in the elderly [39,40].

### 3.2. Diabetic Retinopathy (DR)

DR is a microvascular disease that is clinically diagnosed with the development of structural anomalies in the retinal vasculature leading to the loss of vision. However, multiple investigations have revealed that DR also results in abnormalities of photoreceptor, amacrine, and retinal ganglion cells [41,42]. Increased oxidative stress induced by hyperglycemia, aldose reductase, or inflammation [43] is associated with vascular dysfunction [44], neuronal dysfunction [45], and mitochondrial DNA damage [46], which turns into vascular leakage and neovascularization. Laser surgery and anti-VEGF therapy are currently used to treat DR to regulate the formation of aberrant blood vessels. However, they do not halt the progression of the disease [47]. The present treatments mainly focus on mid- to late-stage DR, where irreversible vision loss is inevitable. The proportion of diabetic individuals who have visual loss could be greatly reduced by combining neuroprotective medicines with novel screening techniques for early stage DR [48].

One of the primary characteristics of retinal neurodegeneration is thought to be an imbalance in neurotrophic factors. Peptides such as brain-derived neurotrophic factor (BDNF), somatostatin (SST), insulin and insulin-like growth factors (IGF), and pigment epithelium-derived factor (PEDF) are shown to improve the neuronal health [49]. These chemicals play a key role in neurovascular connection as well as the growth, differentiation, and maintenance of numerous retinal parts. The retinal pigment epithelium produces PEDF and possesses anti-angiogenic and neuroprotective effects [50]. In addition, PEDF has anti-inflammatory properties and reduces oxidative stress and damage caused by glutamate excitotoxicity [51]. Recent research suggests that retinal neurodegeneration may be caused by the renin-angiotensin system (RAS). Angiotensin II (ATII) is known to mediate oxidative stress, induce angiogenesis, and result in retinal damage [52,53], while RAS is elevated in DR. The effectiveness of neuroprotective medicines in preventing or treating vision loss in DM has not yet been established in humans, despite encouraging preclinical data. Electrospun nanofibers and self-assembled nanofibers are now the two major forms of nanomaterials employed for retinal regeneration. In a study, nanofibers with a diameter of approximately 200 nm were found to more closely resemble the distribution of nerve fibers than those with a diameter of around 1000 nm, facilitating retinal regeneration [54]. The first class of non-viral gene nanocarriers being researched for gene delivery for ocular tissue regeneration is liposome-protamine-DNA (LPD) complexes. They provided higher levels of gene expression while better protecting the plasmid DNA from enzymatic destruction [55]. Translation from animals to humans is frequently challenging due to conceptual and methodological issues, in part because the animal models of human disease are often inaccurately reproduced in humans [56].

### 3.3. Retinal Ganglion Cell Disease

A last link between retinal processing and higher-level visual processing in the midbrain and cortex is provided by retinal ganglion cells (RGCs). Glaucoma and anterior ischemic optic neuropathy are the two main conditions that impact RGCs anterior ischemic optic neuropathy (AION) [57]. Glaucoma is a progressive optic neuropathy characterized by a loss of retinal ganglion cells (RGCs) and typical visual field abnormalities [58]. Although normal tension glaucoma damages RGCs without an increase in intraocular pressure (IOP), it has been demonstrated that ocular hypertension is a significant risk factor implicated in the development and progression of the illness. The most prevalent kind of glaucoma, primary open angle glaucoma, is characterized by RGC axon destruction and consequent loss of RGC cell bodies [59,60].

The precise mechanisms causing axonal damage and hastening RGC mortality are not known. According to a popular notion, elevated IOP may increase the pressure on RGC axons as they leave the eye and enter the optic nerve, which would subsequently cause RGC death [61]. Due to vascular dysregulation, impairments in anterograde and retrograde transport in the RGC axon, metabolic stress, activation of molecular cell death pathways in axons, and neuroinflammation, glaucoma can also arise from conditions other than high IOP [62]. Glaucoma therapy includes surgery or eye drops, which will lower the IOP and reduce the risk of glaucoma [63]. However, not all patients respond to these treatments, and eyedrops have notoriously poor patient adherence [64,65]. RGC death and vision loss can linger even after these therapy methods are effective. Additionally, even with a normal IOP, RGC degeneration advances in patients with normal tension glaucoma [66,67]. As a result, neuroprotective agents should be created to combat secondary RGC degeneration either on their own or in conjunction with IOP reducing techniques. There are many compounds that have been discovered to slow down or prevent the neurodegenerative processes brought on by glaucoma, but they are not very successful. Considering the limitations of conventional therapy, neuroprotective agents should be considered to combat secondary RGC degeneration either on their own or in conjunction with IOP reducing techniques. Additionally, various studies have shown a connection between glaucoma, neuronal cell death, and the increased release of glutamate in the retina [68]. When compared to healthy people, glaucoma sufferers’ vitreous and retinal mounts showed elevated amounts of glutamate and glutamine (a metabolic precursor) and lower levels of the glutamate transporter excitatory amino acid transporter 1 [69]. This further confirms the role of neurotransmitters in the disease and needs to be studied well. According to a study by Huang and team, RNAi therapies can be delivered using lipid-based nanoparticles to achieve high gene silencing efficiencies. RGC-related retinal disorders may one day be treated with RNAi carriers. Other nanocarrier systems comprising of D-α-Tocopherol polyethene glycol 1000 succinate nanoparticles stabilized with Pluronic-F127 were used to deliver curcumin for treating neurodegenerative disorders [70]. This formulation was shown to be successful in lowering RGC loss in two well-known mouse models of optic nerve disease, partial optic nerve transection (pONT) and ocular hypertension (OHT) following topical application as an eye-drop [70,71].

## 4. Neuroprotective Agents

**Figure 2:**
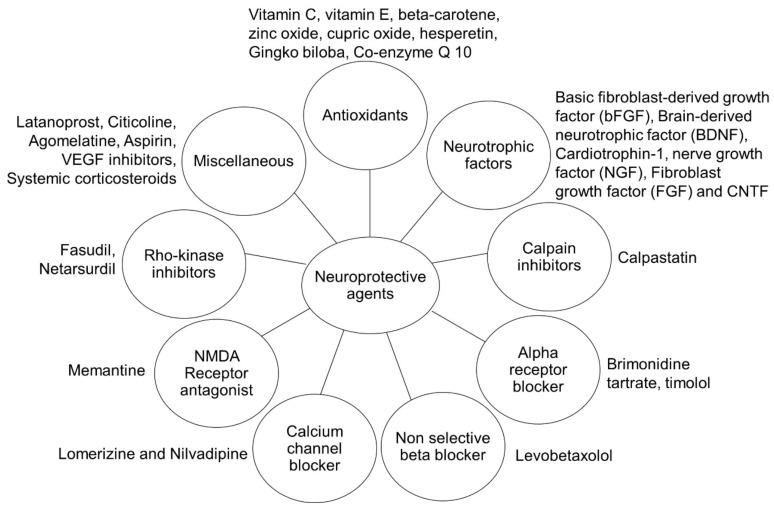
Neuroprotective agents in the treatment of ocular disease.

Numerous neuroprotective compounds have been explored, but only a small number of them have been successful in moving from the laboratory to the clinic (Figure 2). A majority of them, despite encouraging preclinical data, failed to pass the Phase 2 and Phase 3 clinical trials [72]. 

Several antioxidant compounds have demonstrated neuroprotective activity in either in vitro or in vivo models of neuronal cell death or neurodegeneration. For example, vitamin C was found to be effective in treating loss of visual acuity, diabetic retinopathy, and age-related macular degeneration. In vitro assay of vitamin C showed decreased oxidative stress-induced neuronal cell membrane [73]. In a randomized controlled trial, vitamin E showed protection in macular degeneration [74]. β-carotene is a precursor of vitamin A, which essentially functions in many biological processes, including vision. β-carotene increases the glutathione, catalase and superoxide dismutase antioxidant enzyme levels, and eliminates the oxidative stress [75]. In mice exposed to bright visible light, zinc oxide significantly lowered the loss of vision cells. Zinc oxide slowed the progression of age-related macular degeneration in its latter stages [76]. In age-related macular degeneration, cupric oxide has shown promising results by reducing the oxidative damage. Co-enzyme Q10 is known to reduce inflammation and oxidative damage in diabetic retinopathy [36]. Faktorovich et al. showed that bFGF has a neuroprotective effect. However, its usefulness in a therapeutic environment has been limited by the development of serious adverse effects such as retinal revascularization [77]. In numerous animal models, the introduction of encapsulated cells that secrete CNTF into the vitreous has demonstrated the preservation of retinal integrity [78]. To determine if CNTF can enhance photoreceptor function, as measured by visual acuity and visual field sensitivity, in RP patients, a phase 3 clinical trial is now being conducted [79].

A family of cysteine proteases known as caspases was targeted by Leonard et al. for the determination of apoptotic process in a non-specific manner [80]. Eyes of homozygous albino transgenic rats given with the X-linked inhibitor of apoptosis (XIAP) protein showed considerably more intact outer nuclear layers than their contralateral untreated counterparts, demonstrating the protein’s neuroprotective function [80]. The highly specific calpain inhibitor calpastatin peptide was used to pharmacologically block calpain activity in rd1 organotypic retinal explants. This had the effect of reducing photoreceptor cell mortality in vitro following both brief and protracted exposure. These results emphasize the value of calpain inhibitors in delaying or preventing RP [81].

Lack of survival factors secreted by the normal retina in RP also leads to cone death. The finding that rods produce a diffusible component, boosting cone survival in the retinal degeneration animal model, has been corroborated [82]. Injections of the RdCVF protein led to a rise in the number of cone cells and, more importantly, a further increase in the associated ERG in the P23H rat, a model for a common kind of rhodopsin mutation [83]. This makes the protein a possible therapeutic alternative. Additionally, the degree of retinal neuronal damage was reduced, possibly as a result of the PEDF molecule’s anti-oxidative properties [84]. In diabetic rats, injection of systemic and intravitreal insulin was observed to enhance insulin receptor activation and pro-survival signaling [85]. It is thought that BDNF is essential for retinal cell survival. Interestingly, non-diabetic rats also had an increase in dopaminergic neuron cell density after receiving BDNF treatment [86]. In both acute and chronic glaucoma models, therapies that target BDNF receptor activation have been effective in improving RGC survival [87,88]. A distinctive member of the FGF family, fibroblast growth factor (FGF) 21, is crucial for maintaining metabolic homeostasis in diabetes [89]. A long-acting FGF21 analogue was found to be associated with enhanced photoreceptor function and morphology, as well as decreased photoreceptor-derived oxidative stress and retinal inflammation, most recently in the study by Fu et al. [90]. SST and its receptors inhibit VEGF expression to display anti-angiogenic effects [91].

The underproduction of SST in human eyes has been linked to microglial activation and neuronal death, particularly in the GCL [92]. Additional therapeutic targets include nerve growth factor (NGF) and glucagon-like peptide-1 (GLP-1). In the db/db animal model, topical application of GLP-1R agonists (liraglutide) as eye drops was reported to maintain blood-retinal barrier integrity by reducing VEGF overexpression, but its systemic administration revealed notable neuroprotective advantages. Additionally, NGF-containing eye drops have been shown to shield retinal ganglion cells from harm in glaucoma and DR experimental models [92,93]. Telmisartan was shown by Ola et al. to have positive effects (decreasing apoptosis in the retina) in an experiment model where diabetic rats (AT1R) were used [94]. Numerous studies indicate that oxidative-stress likely contributes significantly to the aetiology of DR. It has been demonstrated that giving the flavonoid hesperetin to diabetic mice can reduce the retinal disarray brought on by augmented intercellular gaps in the INL and ONL and prevent the DM-induced possessions of cell inflammation [95].

It is believed that the third-generation alpha-2 adrenergic agonist brimonidine tartrate, which has antiapoptotic characteristics, has neuroprotective effects. [2021] RGC survival was 50% higher in the brimonidine-treated eyes compared to the timolol-treated eyes in a chronic ocular hypertension rat paradigm [82,96]. Brimonidine also reduced the risk of visual field progression compared to timolol in patients suffering from glaucoma. Its potential for neuroprotection is demonstrated by this effect, which is independent of IOP-lowering effects [97]. Latanoprost-prostaglandin analogues have demonstrated neuroprotective effects on glutamate-induced RGC death and axotomy-induced ocular neuropathy in animal models, in addition to significantly lowering IOP [98]. Latanoprost is a glutamate inhibitor and it also inhibits hypoxia-induced apoptosis and functions through cyclooxygenase-2 pathway in order to achieve its neuroprotective benefits [99].

Levobetaxolol, metipranolol, and timolol possess neuroprotective effects by reducing sodium and calcium influx. This influx is linked to the activation of NMDA receptors, which cause ischemia/reperfusion injury [99,100,101]. Fasudil and netarsudil are two rho-kinase inhibitors, which enhance ocular blood flow, halt axonal degeneration, and promote axonal regeneration [102]. Gingko biloba leaf extract with its antioxidant and vasoactive qualities helps to prevent oxidative and apoptosis-mediated degeneration to the optic nerve in glaucoma. In a clinical trial, Gingko biloba extract decreased endothelin-1, which led to vasodilation, decreased plasma malondialdehyde and low-density lipoproteins. This indicates the initiation of an antioxidant response and attenuates the oxidative stress in patients with primary open-angle glaucoma [103]. 

Coenzyme Q10 (CoQ10), a cofactor of the electron transport chain, guards against oxidative stress by maintaining the mitochondrial membrane potential, promoting ATP synthesis, and preventing the production of ROS in neural cells [104,105,106]. CoQ10 shielded retinal neurons from oxidative stress caused by hydrogen peroxide in vitro and NMDA-induced oxidative stress, according to a study by Nakajima [107]. Memantine was thought to be a promising neuroprotective medication for glaucoma, but two randomized, placebo-controlled multicenter trials found no discernible change between patients taking memantine and those taking a placebo in halting the progression of the visual field [108].

Cytidine 5′-diphosphocholine, often known as citicoline, is another intriguing drug candidate for the treatment of glaucoma. A naturally occurring cell-endogenous substance called citicoline serves as an intermediary in the creation of membrane phospholipids like phosphatidylcholine [109]. According to experimental research, citicoline has a neuromodulator effect that could protect RGCs by increasing the synthesis of phospholipids in the CNS [110]. According to Oshitari et al. [110], citicoline has an antiapoptotic effect on mitochondria-dependent cell death mechanisms and plays a supporting function in axon regeneration [111].

Agomelatine, a melatonin analogue that has demonstrated both IOP-lowering and antioxidant capabilities, is currently gaining attention for its pharmacological activity in research [112]. Additionally, CCBs have the therapeutic potential to ameliorate the corrosive processes in open angle glaucoma that are not dependent on IOP. Experimental animals, healthy people, and people with open-angle glaucoma are all affected by CCBs’ general ability to dilate ocular arteries and upsurge ocular blood flow [113]. In vivo investigations on several medications, such as lomerizine and nilvadipine, have produced encouraging neuroprotective outcomes. However, there are issues with CCB-related systemic hypotension because it can exacerbate retinal ischemia by lowering OPP [114]. Anterior ischemic optic neuropathy (AION) makes an optic nerve stroke, causing sudden vision loss. Aspirin, VEGF inhibitors, and systemic corticosteroids are among the treatments for AION. The outcomes are contradictory, and other therapeutic approaches are required [115,116].

## 5. Barriers to Ocular Drug Delivery

**Figure 3:**
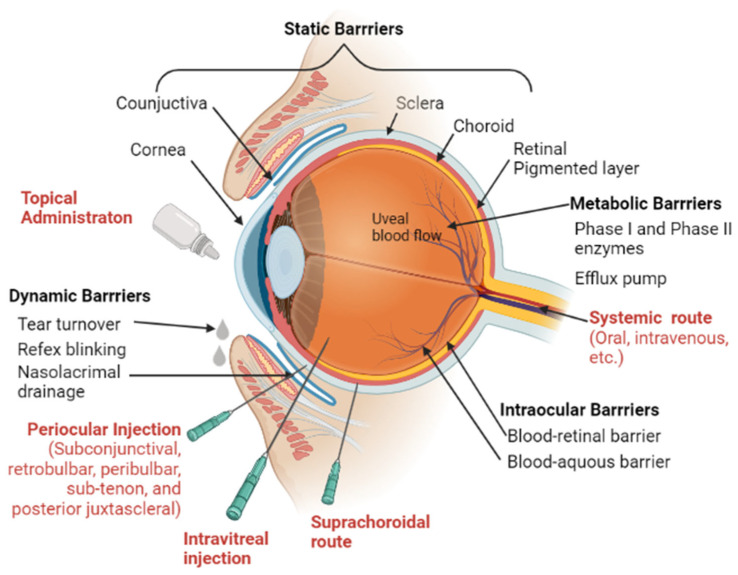
Barriers to ocular drug delivery and routes of ocular drug delivery. Static, dynamic, intraocular, and metabolic barriers to the topical administration of therapeutic agents to the anterior surface of the eye and to the posterior segment are illustrated. Image was created in BioRender.com.

The eye displays a very fine architecture organized in different cell layers, each one deputed to specific functions. It is composed of two segments enveloped by the sclera: the anterior and posterior segments. Diabetic retinopathy, retinal ganglion cell disease, and photoreceptor degenerative disease are the major diseases affecting the posterior eye segment. Drug instillation in the form of eye drops and systemic administration is often ineffective to reach the posterior segment, because of the poor corneal permeability and of the blood retinal barrier, respectively. Preferred routes of drug administration for the treatment of posterior segment diseases include, both intravitreal (IVT) and subretinal. The ocular barriers have a great impact on drug pharmacokinetics and subsequently on the pharmacological effect (109). The ocular anatomy and physiological obstacles are described in Figure 3.

Static barriers: Physical impediments known as static barriers prevent the diffusion of drug molecules from reaching the intended tissues. The cornea and conjunctiva are the two main static barriers in the anterior region. The static barriers of the posterior region of the eye are made up of the sclera, choroid, and retinal-pigmented epithelium (RPE). The corneal tight connections prevent the paracellular diffusion of polar molecules, whereas the transcellular mechanism allows lipophilic medicines to easily diffuse across the lipophilic cornea. Lipid-soluble molecules encounter the hydrophilic stroma as a rate-limiting barrier, which prevents their deeper ocular permeation [117]. Conjunctiva exhibits relatively high paracellular permeability than the cornea for proteins (insulin, molecular weight 5.8 kDa) and peptides (p-aminoclonidine, molecular weight 245.1 Da) due to its larger surface area and leaky vasculature [118,119]. Transcellular pathway plays a higher role in the absorption of lipophilic drugs than hydrophilic drug molecules. Drugs with a log P > 0 are hydrophobic in nature and diffuse the epithelium more readily than those with a log P = 0, which are highly water-soluble and promote stromal diffusion. Although hydrophilic, water-soluble molecules are more readily solubilized in eye drops, their ocular penetration remains a concern. For instance, synthetic cannabinoids are known for reducing IOP and neuroprotective effects. The pharmacokinetics of synthetic cannabinoids (water-soluble derivative, O-2545 and lipid-soluble derivative, O-1812) was compared using microdialysis sampling in an artificially perfused rat eye. This study concluded that lipophilic O-1812 showed better penetration than hydrophilic O-2545 [120]. Lipophilicity of neuroprotectants such as Vitamin E were used in improving the absorption of other hydrophilic drug molecules. Peng et al. utilized the lipophilic nature of Vitamin E to create a diffusion barrier by forcing hydrophilic drug molecules (timolol, dexamethasone 21-disodium phosphate, and fluconazole) to diffuse through the long tortuous path and prolong the drug release [121]. A majority of neuroprotective drugs are lipophilic in nature with fairly high molecular weight and poor water solubility (Table 1). These properties hinder the penetration of molecules in therapeutic concentrations into ocular tissues following topical application. In an attempt to increase the penetration of neuroprotectants, preservatives such as benzalkonium chloride were used to relax the tight intercellular junctions of the corneal epithelium [122]. For example, the ophthalmic solution OMK1 (citicoline 2% + high molecular weight hyaluronic acid + benzalkonium chloride) facilitated the passage of citicoline into the posterior segment tissues such as the retina and the optic nerve head [122]. 

Dynamic barriers: The precorneal and posterior factors are examples of dynamic barriers. Drug dilution is brought on by precorneal variables such tear film and blinking. Drug clearance is influenced by posterior variables such as blood and lymphatic flow. Precorneal tear drainage washes the topically applied drops or solutions within the first 15 to 30 s, significantly shortening the contact time of the drug with the eye and lowering its bioavailability. Conjunctival blood and lymph significantly restrict sodium fluorescein’s ability to go to the inner retinal tissues demonstrating the fickle nature of drug clearance [123]. Some drugs can also alter the normal ocular physiological process. For example, epinephrine can induce tear production, while local anesthetics such as tetracaine can suppress it. Transporters may be amenable to bind and transport specific targeting ligands attached to drug moieties. Efflux and influx transporters also form part of a dynamic barrier. P glycoprotein acts as an efflux transporter for lipophilic drugs [124]. Several neuroprotective agents are identified as substrates of P-glycoprotein (Table 1). CoQ10 concentration in the human retina declines by up to 40% with age. However, its poor aqueous solubility and interaction with multi-drug efflux pump P-glycoprotein (P-gp) expressed in both corneal epithelial cells [125] and RGCs [126] results in low bioavailability following topical administration of the drug. The co-administration of a P-glycoprotein inhibitor, such as D-α-Tocopherol polyethylene glycol 1000 succinate (TPGS), along with CoQ10, has demonstrated neuroprotection against RGCs loss in both in vitro and in vivo mitochondrial-mediated neurotoxicity models following twice daily topical instillation of CoQ10/TPGS micelles [106].

Metabolic barriers: The enzyme systems and transporters are examples of metabolic barriers. Phase I and phase II metabolic enzymes include cytochrome P450, monoamine oxidases, and lysosomal enzymes. The main role in metabolizing enzyme is detoxication of drugs [127,128,129]. The eye also possesses metabolizing enzymes for the prevention or accumulation of the xenobiotics and other substances from the systemic as well as external environment. A variety of esterases such as acetyl, butyrl, and carboxycholine esterases are expressed in ocular tissues [130]. Cholinesterase is expressed in the iris, ciliary body, cornea, lens epithelium, retina, and vitreous humor for drug metabolism. Different esterase classes, i.e., acetylcholine esterase, pseudocholine esterase, butyrylcholine esterase, and carboxyl esterases are expressed in the iris-ciliary body and the corneal epithelium. The expression of these enzymes may enable the development of prodrugs with ester linkages following ocular transport. CYP family enzymes such as aryl hydrocarbon hydroxylase, 7-ethoxycoumarin-O-deethylase, and benzphetamine demethylase are present in the corneal epithelium. β-naphthoflavone (BNF) and barbiturates are easily metabolized during the phase 1 metabolism. Phase 2 metabolizing enzymes such as glutathione S-transferases are present in multiple forms in many tissues. They are responsible for the cellular integrity of the cornea through a complex interplay of drug metabolism and detoxification via conjugation and free-radical scavenging [131]. The reductase activity has been reported in N-oxide, hydroxamic acid, sulfoxide, and nitrocompounds in bovine ciliary body. The amount of cytochrome P-450 in the iris-ciliary body relative to other metabolizing tissue is about 3% of the liver, ~50% of the lungs, and 200% of that present in the cornea [130]. 

Melanin is a macromolecule present in pigmented cells of the eye, such as uveal tract (iris, ciliary body, choroid), and in the retinal pigment epithelium (RPE). The accumulation of drugs in pigmented tissues due to binding with melanin is known for a long time. Melanin can interact with lipophilic drugs and basic drugs, resulting in the alternation of drug disposition [132]. A wide variety of drug categories, including beta-blockers, antibiotics, antidepressants, antimalarials, anesthetics, and sympathomimetics, are known to have melanin-binding drugs. For example, 10–100-fold higher concentrations of timolol was observed in pigmented ocular tissues compared to corresponding albino tissues [133]; however, higher total concentrations of the drug in pigmented tissues did not produce a higher response than in albino animals [134]. Though a majority of neuroprotectants are fairly lipophilic in nature (Table 1), their binding capacity to melanin is yet to be studied. A deeper understanding of the effect of melanin binding on neuroprotectants will broadly benefit drug development.

**Table 1:** Summary of key physicochemical parameters of neuroprotective agents.

Drug	M.W (g/mol)	H Bond Donor Value	H Bond Acceptor Value	Log P	TPSA A˚^2^	P-Glycoprotein Substrate	Log s	Rule of 5	Ref
Memantine	179.3	1	1	2.07	26	Substrate	−3.6	Yes	[135]
Dizocilpine	221.30	1	1	3.14	12	Substrate	-	Yes	[136]
Riluzole	234.20	1	7	3.43	76.4	Substrate	−3.8	Yes	[137]
Brimonidine	292.12	2	3	1.7	62.2	Substrate	−3.3	Yes	[138]
Co enzyme Q10	863.3	0	4	10	52.6	Substrate	−3	Yes	[139]
Citicoline	488.32	3	11	−1.4	214	Substrate	−1.8	Yes	[140]
Flunarizine	404.5	2	3	5.75	6.5	Substrate	−5.4	No	[141]
Lomerizine	468.5	0	7	5.08	34.2	Inhibitor	−4.8	No	[142]
Minocycline	457.5	5	9	0.05	165	Substrate	−2.2	Yes	[143]
Lutein	568.9	2	2	7.9	40.5	Substrate	−5.9	No	[144]
Ginkgo biloba extract	358.6	1	1	1.06	20.2	Inhibitor	-	-	[145]
Vitamin C	176.12	4	6	−1.85	107	Non-Substrate	0.14	Yes	[146]
Vitamin E	430.7	1	2	12.2	29.5	Substrate	−7.6	No	[74]
Palmitoyl-ethanolamide	299.5	2	2	-	49.3	-	-	-	[147]
Melatonin	232.28	2	2	1.6	54.1	Substrate	−3.2	Yes	[148]
Taurine	125.15	2	4	−3.36	88.8	Non-substrate	−0.08	Yes	[149]
Resveratrol	228.24	3	3	3.10	60.7	Non-substrate	−3.5	Yes	[150]
Forskolin	410.5	3	7	1.36	113	Substrate	−2.6	Yes	[151]
Curcumin	368.4	2	6	3.29	93.1	Substrate	−4.8	Yes	[152]
Saffron	977	14	24	-	391				[153]
Timolol maleate	432.5	4	12		183	Substrate	−3.1	Yes	[154]
Azithromycin	749	5	14	4.02	180	Substrate	−3.2	No	[155]
Ampicillin	394.4	3	6	1.35	138	Substrate	−2.8	Yes	[156]
Ofloxacin	361.4	1	8	−0.39	73.3	Substrate	−2.4	Yes	[157]
Fluorometholone	376.5	2	5	2	74.6	Substrate	−4.4	Yes	[158]
Voriconazole	349.31	1	8	1	76.7	Substrate	−3.6	Yes	[159]
Triamcinolone acetonide	462.5	1	8	2.31	99.1	Substrate	−2.7	Yes	[160]
Natamycin	665.7	7	14	1.1	231	Substrate	−3.4	No	[161]
Spironolactone	416.6	0	5	2.78	85.7	Substrate	−5.3	Yes	[162]
Fluconazole	306.27	1	7	0.5	81.6	Non-substrate	−2.3	Yes	[163]
Moxifloxacin	401.4	2	8	2.9	82.1	Substrate	−3.4	Yes	[164]
Propranolol hydrochloride	295.80	3	6	−0.45	41.5	Substrate	−3.5	Yes	[165]
Cysteamine	77.15	2	2	0.1	27	Substrate	−2.4	No	[166]
Cyclosporin A	1202.6	5	12	1.4	279	substrate	−0.5	Yes	[167]
Dexamethasone	392.5	3	6	1.83	94.8	Substrate	−3.9	Yes	[168]
Ketorolac tromethamine	376.4	5	7	2.28	146	Non-substrate	−2.7	Yes	[169]

TPSA: Topological polar surface area, LogS: Aqueous solubility.

## 6. Nanocarrier Systems in the Delivery of Neuroprotective Agents

**Figure 4:**
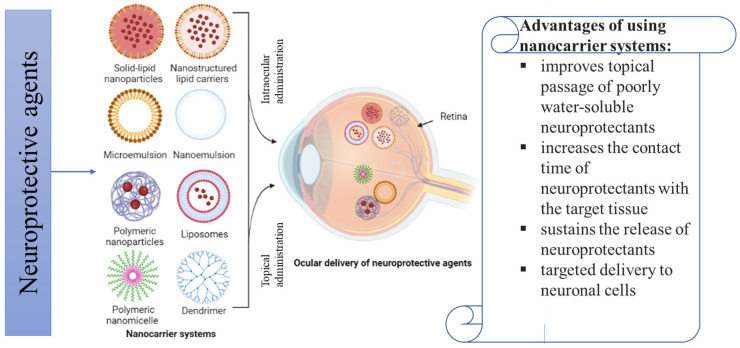
Nanocarriers systems for the ocular delivery of neuroprotective agents in the treatment of ocular neurodegenerative diseases. Image was created in BioRender.com.

In ophthalmic drug delivery, the mode of drug administration is considered as a bottleneck for the transition from experimental findings in the lab to its testing in clinical trials. The beneficial effects of neuroprotective agents are limited by drug bioavailability in the retina. Systemic administrations of neuroprotective agents for treating ocular disorders could adversely affect the endocrine and/or central nervous systems, while topical administrations as eye drops require a high frequency of administration to maintain therapeutic drug concentration in the retina. The use of various nanocarrier systems can increase drug bioavailability and improve the delivery kinetics of neuroprotective agents through sustained release of molecules at the target sites. Different nanocarriers, such as polymeric NPs, solid-lipid nanoparticles (SLNs), liposomes, polymeric micelles, and dendrimers, have so far shown promise in overcoming most ocular barriers and improving medication delivery to the back of the eye (Figure 4). They are useful for directing medications to the retina because of their favorable physicochemical characteristics such as tiny size, adaptable surface qualities, higher in vivo stability, increased the permeability of drugs across biological membranes to achieve higher bioavailability, and ability to provide sustained release at the target site. In this section, nanocarrier systems used in the delivery of neuroprotectants to the eye will be discussed. 

### 6.1. Lipid Nanoparticles

Lipid nanoparticles, such as SLNs and nanostructured lipid carriers (NLCs), have a lipid core with only a few nanometers in size, and they are stabilized by a surfactant layer. Both lipophilic and hydrophilic drugs can be loaded into the lipid core. NLCs are considered advantageous compared to SLNs as they hold both liquid and solid lipids. The advantages of NLCs include higher drug-loading capacity, and better stability and release [170]. The mucoadhesive qualities of lipid nanoparticles let them interact more effectively with the ocular mucosa and extend the drug’s duration in the eye. Lipid nanoparticles offer a number of benefits over other colloidal carriers, including the potential for controlled drug release, long-term stability, the capacity to encapsulate hydrophilic or lipophilic medicines, a high drug loading rate, and the absence of biotoxicity [171]. Lipid nanoparticles have exceptional biological qualities that make them suitable as carriers for drugs belonging to BCS classes II and IV. Early research on curcumin SLNs with PEGylated SLNs indicated positive outcomes in macular degeneration [172]. Shah et al. developed and optimized lutein-loaded SLNs for the treatment of AMD [173]. The ex vivo evaluation revealed that the optimized formulation did not cause any damage to the cornea and sustained the drug release for up to 8 h in simulated tear fluid [173]. In a different study, Noha et al. formulated brimonidine NLCs and SLNs. The results showed that brimonidine NLCs improved corneal permeability, enhanced the drug localization in the anterior ocular chamber, and enhanced ocular hypotensive effect of brimonidine [174]. Liu et al. reported that the quercetin-loaded SLNs showed the highest flux across the cornea after 24 h. In addition, quercetin-loaded SLNs could efficiently protect the cornea and RGCs from H_2_O_2_-induced oxidative damages [175]. Likewise, genistein encapsulated NLC was produced by Zhang et al. This study concluded that NLCs extended the precorneal clearance of genistein and thus increased its corneal penetration and bioavailability [176]. Surface-modified lipid nanoparticles were also reported in literature for the delivery of neuroprotectants. Surface-modified particles show prolonged contact time with the cornea and thus improve the ocular bioavailability of drugs [177]. Attama et al. reported that surface-modified SLNs is an efficient way of improving ocular bioavailability of timolol hydrogen maleate [178]. Dang et al. showed that latanoprost-loaded PEGylated SLNs improved the drug loading in contact lens and sustained the drug release for use in glaucoma therapy [179]. The residence time of lipid nanoparticles can be improved by dispersing them in in situ gelling polymers. Wadetwar et al. showed that bimatoprost nanoparticles loaded pH-sensitive in situ gel sustained the drug release and can offer improved management of glaucoma [180]. Similar results were also observed with triamcinolone acetonide-loaded SLNs suspended in in situ gel formulations. Triamcinolone acetonide -loaded SLNs and in situ gel formulations showed an improved pre-corneal residence time and a sustained delivery of the drug into the anterior and posterior segment ocular tissues [181]. Yu et al. developed a hybrid nanostructured lipid carrier/dual pH- and thermo-sensitive hydrogel for ocular delivery of quercetin. Due to longer precorneal retention time, the bioavailability of quercetin in the hydrogel group was increased 4.4-fold compared to eye drop [182].

SLNs of dexamethasone were created and their toxicity and ocular permeability were examined. The corneal permeability and residence time of dexamethasone was improved. The 8 h prolonged release of natamycin from SLNs had a substantial impact and higher corneal penetration [183]. Contrary to drug suspension, the cationic nano-lipoidal formulation of triamcinolone acetonide demonstrated a 2-fold increase in the corneal penetration. Moxifloxacin and lipid-polymer hybrid nanoparticles of hyaluronic-acid were created by Dan Liu. The mean residence time (MRT) and area under the curve (AUC_0–6h_) of HA-LCS-NPs were up to 6.74-fold and 4.29-fold higher than those of the commercial product, according to an in vivo precorneal retention investigation in rabbits [184]. Although published data show the potential of lipid nanoparticles in preclinical studies, lack of extended clinical trials in humans remains a drawback. Further, the long-term toxicity of lipid nanoparticles on retinal cells should be evaluated.

### 6.2. Liposomes

**Figure 5:**
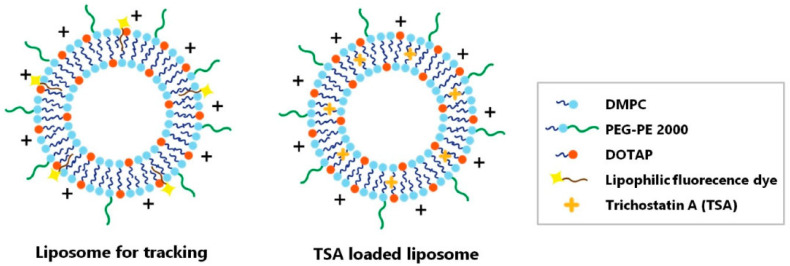
Schematic illustration of liposome for tracking and trichostatin A-loaded liposome. Two liposome particles were synthesized. (Left) The liposome was synthesized for the purpose of tracking the location of the drug when it was administered to the vitreous cavity. (Right) The liposome was loaded with trichostatin A and synthesized for the purpose of treatment. DMPC = 1,2-dimyristoyl-sn-glycero-3-phosphocholine; PEG 2000-PE = 1,2-distearoyl-sn-glycero-3-phosphoethanolamine-N-[methoxy(polyethylene glycol)-2000]; DOTAP = 1,2-dioleoyl-3-trimethylammonium propane. Reproduced with permission from Sung et al. [190].

**Figure 6:**
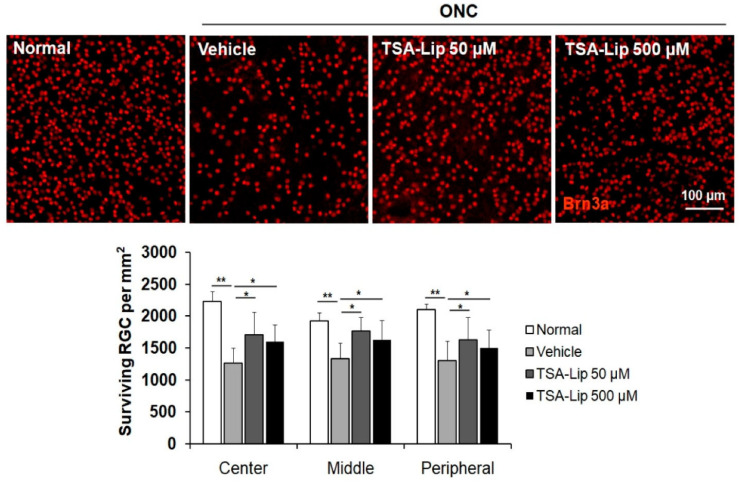
Effect of trichostatin A-loaded liposomes on RGC survival after ONC. Retinal flat mounts are shown for the uninjured normal retinas, vehicle-injected retinas, and retinas injected with 50 μM and 500 μM trichostatin A-loaded liposomes (*n* = 5 retinas per group). A quantitative analysis of RGC survival in center, middle, and peripheral zones of each retinal quadrant is also shown as a graph. Trichostatin A-liposomes 50 µM = liposomes loaded with 50 µM trichostatin A; trichostatin A-liposomes 500 µM = liposomes loaded with 500 µM trichostatin A. Scale bar = 100 µm; ** *p* < 0.001, compared to the uninjured normal retina; * *p* < 0.05 compared to the vehicle-injected retina; one-way ANOVA with post hoc LSD test. Reproduced with permission from Sung et al. [190].

Liposomes are nano- to micro-sized vesicles enclosed by a phospholipid bilayer made up of phospholipids and cholesterol. They are excellent vehicles for drug delivery because they can encapsulate both hydrophilic and hydrophobic drugs [185]. The phospholipids utilized to create liposomes, such as phosphatidylserine and soybean phosphatidylcholine, are analogous to the lipids on the cell membrane, and hence liposomes often exhibit minimal immunogenicity and low toxicity [186,187]. Liposomal systems significantly extend the duration of therapeutic activity and increase the drug’s concentration in the posterior segment. In the last five years, a number of liposomal methods have been investigated for drug delivery to the posterior eye [188]. Lutein liposomes were formulated and characterized by Ibrahim et al. using the thin film hydration method. Lutein liposomes showed an antioxidant effect on ocular tissues in cisplatin-induced retinal injury in rabbits. Electroretinogram (ERG) in rabbits depicted a reduction in ERG waves and an increase in all parameters of Comet assay. Further, it was concluded that the liposomal lutein administration could prevent the detrimental effects of cisplatin on the retina, while avoiding the use of any artificial chemicals [189]. A study conducted by Sun et al. evaluated the neuroprotective effects of intravitreally injected trichostatin A-loaded liposomes in a mouse model of optic nerve crush injury. The result surprisingly reduced reactive gliosis and RGC apoptosis and increased RGC survival [190]. In this study, two-liposomal formulations were prepared, one formulation as tracker and the other one for the purpose of treatment (Figure 5). Liposomes were found in the inner retina with fluorescence lasting for up to 10 days after the intravitreal injection. This study reported a reduction in reactive gliosis, RGC apoptosis, and increased RGC survival in a mouse model of ONC [190] (Figure 6). All of this evidence collectively suggests the effectiveness of liposomes over conventional formulation in delivering neuroprotective agents to the eye.

Drugs like berberine hydrochloride and chrysophanol are considered as potential molecules due to their antioxidative, antiangiogenic, and anti-inflammatory effects. Lai et al. prepared a compound liposomal system that entrapped berberine hydrochloride and chrysophanol simultaneously using the third polyamidoamine dendrimer as a carrier. The liposomes exhibited appreciable cellular permeability in human corneal epithelial cells and enhanced bio-adhesion on rabbit corneal epithelium. Moreover, coated liposomes greatly improved berberine hydrochloride bioavailability and exhibited protective effects in human retinal pigment epithelial cells and rat retinas after photo oxidative retinal injury [191]. All of this evidence collectively suggests the effectiveness of liposomes over conventional formulation. 

### 6.3. Polymeric Nanoparticles

Polymeric nanoparticles are created using a variety of biocompatible natural and synthetic polymers [192]. Polylactide (PLA) and poly(lactic-co-glycolic acid) (PLGA) are the most commonly utilized synthetic polymers in the preparation of polymeric nanoparticles. Additionally, natural substances such chitosan [193], hyaluronic acid [194], and cyclodextrin [195] have been used in their preparation. Polymeric nanoparticles are known to have a good ability to deliver drugs to the posterior region of the eye. In a recent study, melatonin-ethylcellulose nanocapsules were prepared and evaluated for antiapoptotic and neuroprotective effects in a retinal degenerative model induced in albino rabbits. Higher penetration of melatonin across the cornea was reported in ex vivo and in vivo tests. Further, melatonin when loaded into ethylcellulose nanocapsules showed an enhanced neuroprotective effect when compared to a solution of melatonin [196]. Memantine, a neuroprotective agent approved by the FDA for Alzheimer’s disease treatment, is known for its ability to prevent RGC death in glaucoma. However, the delivery of memantine to the back of the eye in a safe and effective manner is a great challenge. In a study by Sánchez-López et al., memantine-loaded PLGA-PEG nanoparticles were investigated for efficacy in a glaucoma model. Memantine nanoparticles were found to be well-tolerated when administered topically in a rodent model with ocular hypertension memantine nanoparticles and significantly reduced RGC loss [197]. Davis et al. reported a nanocarrier system of curcumin-loaded Pluronic-F127 stabilized D-α-Tocopherol polyethene glycol 1000 succinate nanoparticles. In vitro studies in R28 cells showed protection against cobalt chloride induced hypoxia and glutamate induced toxicity. Further studies in glaucoma-related in vivo models of ocular hypertension and partial optic nerve transection showed a significant reduction in RGC cell loss compared to controls [70] following topical application of curcumin-loaded nanoparticles twice a day for three weeks. Sustained release of drugs from PLGA nanoparticles can be further enhanced by dispersing them in polymeric hydrogels. Yang et al. reported a novel hybrid polyamidoamine dendrimer hydrogel/PLGA nanoparticle for co-delivery of brimonidine and timolol maleate. This formulation demonstrated enhanced drug bioavailability and sustained IOP reduction for a long duration. Furthermore, this formulation can greatly reduce dosing frequency of topical formulations, thus, improving long-term patient compliance and reducing enormous societal and economic costs [198]. Sharma et al. developed brimonidine tartrate loaded PLGA-vitamin E-tocopheryl polyethylene glycol 1000 succinate nanoparticles and suspended them in a thermosensitive gel for the management of glaucoma. The nanoparticles improved precorneal residence time without causing eye irritation, as well as sustaining the release of brimonidine tartrate through the cornea [199].

Chitosan is a well-known mucoadhesive polymer due to the presence of positively charged amino groups. Chitosan nanoparticles increase the pre-ocular residence time of drugs due to interaction of with the negatively charged residues of sialic acid in the conjunctival and corneal mucosa. In vitro permeation in rabbit’s eye, particularly in sclera, revealed adequate permeation and activity of moxifloxacin and betamethasone loaded chitosan–dextran nanoparticles [200]. Dandamudi et al. developed triamcinolone acetonide-loaded chitosan-coated PLGA NPs for topical administration to the eye in order to treat acquired retinal vasculopathies [201]. The mucoadhesive properties of chitosan can be synergized with hyaluronic acid, a natural polysaccharide. Wadhwa et al. developed hyaluronic acid-modified chitosan nanoparticles-loaded with timolol and dorzolamide, and results suggest that hyaluronic acid potentially enhanced the mucoadhesiveness and efficiency of NPs and may be a promising carrier for ocular drug delivery [202]. These findings clearly indicate the potential of polymeric nanocarriers in the treatment of ocular neurodegenerative diseases.

### 6.4. Nanoemulsions and Microemulsions

Nanoemulsions are defined as “a biphasic dispersion of two immiscible liquids, such as water in oil (W/O) or oil in water (O/W) droplets” [203]. Both hydrophilic and hydrophobic drugs can be contained within the emulsion droplets, which can serve as a drug reservoir. These homogeneous thermodynamically stable systems are less viscous, making them suitable for topical application. The precorneal residence time can be improved by using cationic o/w nanoemulsion as the ocular surface is negatively charged [204]. Although nanoemulsions have many benefits, they have some drawbacks such as the potential to irritate eyes and impair vision after instillation. Through careful selection of surfactants and co-surfactants employed in the preparation of nanoemulsions, it is possible to lower the toxicity and interfacial tension of nanoemulsions [205]. The surfactant or co-surfactant must not irritate the ocular tissues and should not be harmful. In a study by Garg et al., tacrolimus was entrapped in nanoemulsion that showed good penetration through the cornea and thus improved the intraocular concentration of tacrolimus required for managing uveitis [206]. An interesting aspect of this study was that the oils used for preparation of nanoemulsions had natural anti-inflammatory properties, which potentiated the effect of tacrolimus through synergistic action. Surfactants included in NEs have the ability to suppress P-gp function in epithelial cells [207]. The efflux pumps (P-gp and MDR) in the eye could be targeted with NEs so as to increase the bioavailability and permeability of drugs that are substrates of efflux pumps. The selection of appropriate NE formulation ingredients helps in effectively evading ocular barriers and also provides sustained and targeted drug delivery [208]. Chaemin et al. formulated leutin nanoemulsion using isopropyl myristate, triacetin, Tween 80, and ethyl alcohol to improve its solubility/permeability for use in the treatment of macular degeneration. Lutein-loaded nanoemulsion showed a remarkable sustained release of the drug for 7 days [209]. Liu et al. formulated quercetin nanoemulsion for efficient protection of cornea and retinal ganglion cells from H_2_O_2_-induced oxidative damages [175].

Microemulsion is a dispersion of water and oil that is stabilized by a surfactant or co-surfactant to reduce the interfacial tension. They are clear in appearance, and thermodynamically stable with a small droplet size (∼100 nm). Microemulsion because of its intrinsic structure and properties have a high drug dissolving capacity. An oil-in-water type of microemulsion with the presence of surfactant and co-surfactant is able to increase membrane permeability in corneal drug delivery [177]. They are generally low in viscosity and able to deliver drugs in a sustained manner, thus increasing the overall absorption capacity. The increased permeability and sustained release of drugs makes this system an attractive vehicle for ophthalmic drugs. Apart from having a low surface tension, they have a good spreading ability, thus allowing the drug to spread on the cornea and mix well with the precorneal fluid. This improves the contact time of drugs with the corneal epithelium [210]. Timolol malate microemulsion formulated by Wi and his coworkers showed a marked reduction in intraocular pressure and lasted for 12 h as compared to aqueous eye drop that lasted for only 5 h. In vivo reduction of intraocular pressure revealed a similar efficacy for once daily dosed 0.3% timolol maleate microemulsion formulation and 0.5% timolol maleate aqueous formulation. The dose reduction with timolol maleate microemulsion is helpful in reducing cardiovascular side effects that are generally reported with timolol maleate eye drops [211]. Both nanoemulsions and microemulsions are found to be very promising vehicles in drug delivery. They can be used to overcome the problems of neuroprotectants such as stability, solubility, absorption, and permeation across various ocular tissues.

### 6.5. Polymeric Nanomicelles

**Figure 7:**
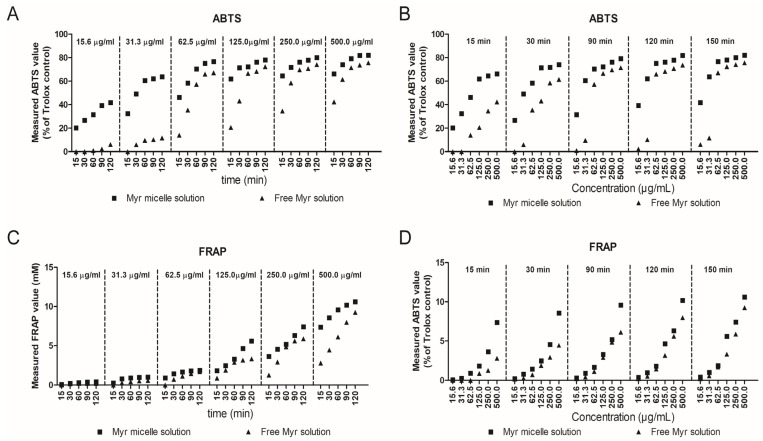
Measured antioxidant characterizations using 2,2′-Azinobis (3-ethylbenzothiazoline 6-sulfonate) (ABTS) free-radical scavenging assay. Measured ABTS values of the free myricetin and myricetin micelles with different incubation times as functions of concentration (**A**) and different concentrations as functions of time (**B**); Measured FRAP values of the free myricetin and myricetin micelles with different incubation times as functions of concentration (**C**) and different concentrations as functions of time (**D**). Reproduced with permission from Sun et al. [219].

Polymeric nanomicelles are the amphiphilic molecules which can self-assemble into organized supramolecular structures. The inner core of polymeric micelles is hydrophobic, while the outer shell is hydrophilic in nature [212]. Micelles are small in size with hydrophilic makeup. These properties facilitate their passage through the corneal barriers and sclera’s hydrophilic pores with ease [213]. In addition to serving as inert nanocarriers for drugs, polymer nanomicelles have the potential to increase the drug’s bioavailability by blocking efflux transporter proteins with polymeric surfactants. Polymeric nanomicelles prepared using amphiphilic block copolymers can improve the solubility and stability of hydrophobic pharmaceuticals [214], prolong drug retention on the surface of the eye, improve corneal permeability [215], induce bioadhesion, and alter drug release [71]. Many nanomicellar preparations containing active ingredients have been reported to treat eye problems [216]. Myricetin exhibits diverse biological and pharmacological properties, including in ophthalmology. Myricetin’s antioxidant activity is shown to be useful in treating eye diseases, especially corneal diseases such as dry eye, which contribute to oxidative stress [217]. Despite various pharmacological benefits and a good safety profile, myricetin lacks sufficient water solubility and aqueous stability in order to be formulated into eye drops [218]. Sun et al. encapsulated myricetin in a polyvinyl caprolactam–polyvinyl acetate–polyethylene glycol graft copolymer polymeric micelles to increase its aqueous solubility, stability, and corneal permeability to promote its efficacy in eye disease treatment. Micelles significantly enhanced myricetin’s aqueous solubility and chemical stability. Furthermore, myricetin micellar ophthalmic solution significantly improved in vitro cellular uptake, in vivo corneal permeation and significant improvements in the in vitro antioxidant activity and in vivo anti-inflammatory efficacy of myricetin [219]. The antioxidant activity measured using 2,2′-azinobis (3-ethylbenzothiazoline 6-sulfonate) (ABTS) free-radical scavenging assay showed an increase in the activity with increasing concentration/incubation time in both the encapsulated myricetin in micelles and the free myricetin solution. However, encapsulated myricetin in micelles exhibited better antioxidant activity compared to free myricetin solution at varying concentrations and incubation times. A similar trend was reported for ferric-reducing antioxidant potential (FRAP) assay evaluation (Figure 7).

Li et al. have used Soluplus^®^ micelles, polyvinyl caprolactam–polyvinyl acetate–polyethylene glycol graft copolymer (PVCL–PVA–PEG), to improve the solubility and stability of resveratrol [220]. Resveratrol micelles promoted cell proliferation in both short- and long-term cytotoxicity tests with no evidence of cytotoxicity. Resveratrol micelles showed higher in vitro passive permeation/cellular uptake and in vivo corneal permeation as compared with free resveratrol suspension solution. Additionally, resveratrol micelles showed better in vivo corneal wound healing and inhibition of anti-inflammation mediators. These studies suggest that polymeric micelles could be used for enhancing the antioxidant properties by improving the solubility and stability of resveratrol.

### 6.6. Dendrimers

Dendrimers have gained attention because of their adaptability, and multi-branched characteristics. Dendrimers are globular, branched nanostructured polymers that resemble a tree-like structure [221]. Drugs may be conjugated by covalent connections or trapped in the dendrimer network through ionic interactions, hydrophobic interactions, or hydrogen bonds. Dendrimers based on poly(amidoamine) (PAMAM) polymers have received tremendous attention in drug delivery due to ease of manufacturing [222]. Dendrimers may have functional groups that are neutral, negatively charged, or positively charged at the end of their branches. Dendrimer hydrogel was prepared for simultaneous delivery of brimonidine and timolol maleate by Holden et al. [223]. As compared to eye drop formulations, dendrimer hydrogel significantly increased the human corneal epithelial cells uptake and bovine corneal transport for both drugs [223]. Polyguanidilyated translocator dendrimers have been formulated by Durairaj et al. and used as delivery for gatifloxacin. This study concluded that the dendrimer-based formulation of gatifloxacin should improve drug solubility, permeability, and anti-bacterial activity against methicillin resistant *S. aureus*. Further, in vivo administration of gatifloxacin-loaded dendrimer eye drop formulation in New Zealand white rabbits resulted in higher tissue concentrations of the drug in conjunctiva and cornea, and sustained levels of gatifloxacin was observed in aqueous humor and vitreous humor [224]. The results of preclinical studies involving dendrimers are very encouraging, however; they should be replicated in further studies in humans.

## 7. Limitations of Nanocarriers for Ocular Applications

Researchers and regulatory agencies encounter numerous difficulties while developing drug-loaded nanocarriers. Robust characterization techniques, scalable optimization strategies, safety requirements, and stability maintenance are required to meet these problems. The formulation process of nanocarriers and subsequent clinical application may run into several obstacles. Surfactants that are added to nanocarrier formulations to aid in dispersion frequently obstruct traditional characterization techniques. Physiological factors including temperature, pH, and ionic strength have a significant impact on the physicochemical properties of nanocarriers, such as charge and hydrodynamic diameter [225]. After injection, the binding of nanocarriers to plasma proteins may modify the distribution and clearance of nanocarriers and entirely alter the drug release profile in physiological fluids. Moreover, changes in polydispersity brought on by contact with bodily fluids may significantly affect the toxicity and biocompatibility of a substance. The safety of nanocarriers in drug delivery systems (DDSs) is one of the biggest challenges associated with this technology. Various studies have examined the safety and toxicity of nanocarriers on the human body. Size, shape, surface charge, delivery route, and drug dose were all found to be linked to the toxicity of nanocarriers. 

As nanocarriers interact with body tissues and biological fluids, it is likely that their nanoscale dimensions will cause toxicity. In order to fully characterize nanocarriers in vivo, it is advised that cytotoxicity studies be carried out [226,227,228,229,230]. Acute nanotoxicity investigations commonly use cell culture models because of their affordability and simplicity. Unfortunately, due to low cellular viability, they cannot be used to assess the results of persistent toxicological exposure. Additionally, there has not been enough research done on the toxicological effects of repeated exposure of tissues to nanocarriers. In addition, a lot of nanocarrier systems, such as peptides and nucleic acids, may cause immunogenic reactions that have major adverse effects or even anaphylactic shock. According to a prior investigation, nanocarriers are susceptible to stability issues after long-term storage that could affect their effectiveness and toxicity. The production of nanocarriers on a large scale requires careful monitoring of exposure levels and any potential effects. The achievement of a positive safety profile will be aided by adequate control over the nanocarrier manufacturing process. Previously, novel toxicological techniques such as particokinetics and multiparametric analysis were reported. For the toxicological assessment of nanocarriers, there is no standardized approach accepted worldwide. The safety implications of nanocarriers should be taken into account, and size, surface charge, and solubility can be utilized to forecast the toxicity of nanocarriers, according to several international standard-setting organizations. 

Another issue with nanocarrier systems is regulatory restrictions. The usage of nanocarriers is governed by the FDA, the European Medicines Agency (EMA), and other regulatory authorities including the Center for Drug Evaluation and Research (CDER) [230]. Only 21 nanocarrier compositions have received approval in the past 30 years. It appears that the lack of established and biorelevant rules for characterization and quality control makes it difficult to move nanocarriers from the bench to the bedside. For instance, developing a general in vitro release technique for nanocarriers is very challenging. Particularly given that there is not a lengthy history of acceptance in the literature, there is a great need to establish and validate fresh, standardized techniques for the safety and characterization of nanocarriers. However, regulatory agencies should assess nanocarrier-based OTC differently as it is difficult to obtain adequate batch-to-batch repeatability because of the polydispersity issues. In addition, numerous large-scale process variables need to be regulated, including solvents, temperature, pH, surfactants, and polymer-to-drug and lipid-to-drug ratios [231,232].

## 8. Conclusions and Future Perspectives

Despite the great prevalence of retina diseases and the complexity of the mechanisms involved, neuroprotective approaches provide optimism that functional vision can be maintained till the end of life, thereby promoting independence and quality of life. However, the failure to optimize dosing required to effectively treat the neurodegeneration in the eye hinders their translation from research laboratory to patient care. In addition, the effectiveness of neuroprotective agents to target ocular neurodegenerative illness is limited due to their inability to effectively cross the blood retinal barrier, short in vivo half-lives, and poor pharmacokinetics. The use of nanocarriers for intraocular administration of neuroprotective agents could be a viable option to overcome these limitations. Nanocarrier systems can effectively deliver both hydrophobic and hydrophilic drugs by entrapping them inside the cavity or through conjugation of drugs using covalent linkages. Several studies have reported that the use of nanocarrier systems, such as nanoemulsions, nanomicelles, dendrimers, polymeric nanoparticles, lipid nanoparticles, and liposomes, enhance the permeability of neuroprotective agents into the eye as well as increase its retention time, thus providing greater efficiency for treating the disease. The reported preclinical studies of nanocarriers provide a hope for safe and effective drug delivery for treating ocular neuroprotective diseases. Nanocarriers can be administered through less invasive techniques and thus can ease the burden for patients as well as healthcare professionals. A better understanding of the interaction of nanoparticles with ocular tissues will contribute to a more successful design of nanoparticles that can efficiently evade the eye’s anatomical and physiological barriers. Further research is needed to thoroughly understand the safety, reproducibility, and scale-up of nanocarriers for reaching clinical trials. Overall, the combination of neuroprotective agents and nanocarriers constitutes a breakthrough toward a next-generation treatment of ocular neurodegenerative diseases.

## Data Availability

Not applicable.
